# Burn Scar Revision With Tissue Expansion, Long-Pulsed 1064-nm Neodymium-Doped Yttrium Aluminum Garnet (Nd:YAG) Laser, and Microneedling: A Case Report

**DOI:** 10.7759/cureus.96228

**Published:** 2025-11-06

**Authors:** Vaibhav Varma, Jessica Routhier, Chantal Wahba, Kimiya Taji, Shay B Dean

**Affiliations:** 1 Plastic and Reconstructive Surgery, Dean Plastic Surgery Associates Inc., Los Angeles, USA; 2 Surgery, St. George's University School of Medicine, St. George, GRD; 3 General Surgery, Sutter Roseville Medical Center, Roseville, USA

**Keywords:** burn scar, laser treatment, microneedling in scar management, plastic and reconstructive surgery, scar revision, tissue expander

## Abstract

Therapeutic heat devices can cause iatrogenic burns that lead to infection, grafting, and disfiguring scars. A 56-year-old woman sustained full-thickness burns of the left flank/posterior trunk after postoperative application of microwaved saline hot packs. The wounds became infected and necrotic, requiring antibiotics, debridement, and split-thickness skin grafting. Two years later, she presented with a hypertrophic, hyperpigmented ~20×10 cm graft scar and contour deformity. We implemented a staged plan: placement of two tissue expanders with serial fills over ~8 weeks; expander removal with complete scar excision and tension-free closure using the expanded native skin; adjunct scar therapies including multiple sessions of long-pulsed 1064-nm neodymium-doped yttrium aluminium garnet (Nd:YAG) laser and microneedling; and a focused office scar-revision excision (6×3 cm) for residual margin hypertrophy. Objective assessment was done with the Modified Vancouver Scar Scale (MVSS), total score improved from ~10 (pigment 3, vascularity 2, pliability 3, height 2) to ~3 at 23 months (pigment 1, vascularity 1, pliability 1, height 0). No expander-related complications occurred. This case shows that for extensive trunk graft scars, staged tissue expansion provides like-for-like, sensate skin to restore contour, while 1064-nm laser and microneedling further improve scar vascularity, thickness, pliability, and color with low morbidity.

## Introduction

Our patient’s initial surgery, during which the saline hot pack injury occurred, was performed in Tehran, Iran. Subsequent treatments were undertaken there and later in the United States for burn management and reconstruction. This context aligns with reports of increased complications among patients who undergo cosmetic surgery abroad, with substantially higher complication rates for patients outside the United States, with risks including pain, infection, delayed wound healing, and even death [1]. Accordingly, caution is advised when considering medical procedures abroad.

Burns involve injury to the epidermis, dermis, and/or subdermal tissue, classified by escalating degrees of severity from superficial to full thickness. Disruption of the epidermal barrier increases susceptibility to infection, often contributing to substantial morbidity and mortality [2]. Burns may result from a variety of iatrogenic and non-iatrogenic causes. Among postoperative patients, hot packs have emerged as a notable source of iatrogenic burns. Comparative studies of therapeutic injury mechanisms have shown that hot packs are the leading cause of postoperative iatrogenic burns, surpassing radiant heat and electric heating pads [3].

Burn scar contractures can severely affect a patient’s quality of life, causing pain, pruritus, disfigurement, and restricted motion. Surgical excision and release of contractures with grafting or flap coverage is often necessary to restore function; however, surgery alone has limitations, including donor site morbidity and a high risk of scar recurrence [4]. When large surface areas are involved, tissue expansion can generate adjacent, like-matched skin for reconstruction, minimizing color/texture mismatch and optimizing contour [5-7]. In recent years, a variety of non-surgical interventions have been used alongside surgery to optimize scar outcomes, including laser treatments and percutaneous needling techniques. Vascular-targeting lasers (e.g., long-pulsed 1064-nm neodymium-doped yttrium aluminum garnet (Nd:YAG)) can reduce erythema and thickness, while microneedling (also known as percutaneous collagen induction) promotes dermal remodeling with minimal downtime [8-12]. We present a case in which these modalities were combined in a stepwise fashion to treat a severe post-burn trunk scar. This case highlights the use of tissue expansion to facilitate scar revision, followed by laser therapy and microneedling to further improve the scar’s appearance and pliability.

## Case presentation

A 56-year-old female patient with no significant comorbidities presented with a history of burns sustained to her left flank and posterior trunk after a liposuction procedure that took place in Tehran, Iran, in 2019. The patient suffered from full-thickness burns after the postoperative application of microwaved saline hot packs. Subsequently, the burn wounds became infected, requiring antibiotics, and eventually necessitated surgical debridement and skin grafting. The resulting graft scars caused discomfort and limited the flexibility of the skin in the area. They had also negatively impacted the patient's self-image and psychological well-being, prompting her to seek plastic surgery evaluation in January 2022 for possible scar revision for both functional improvement and cosmetic enhancement.

**Figure 1:**
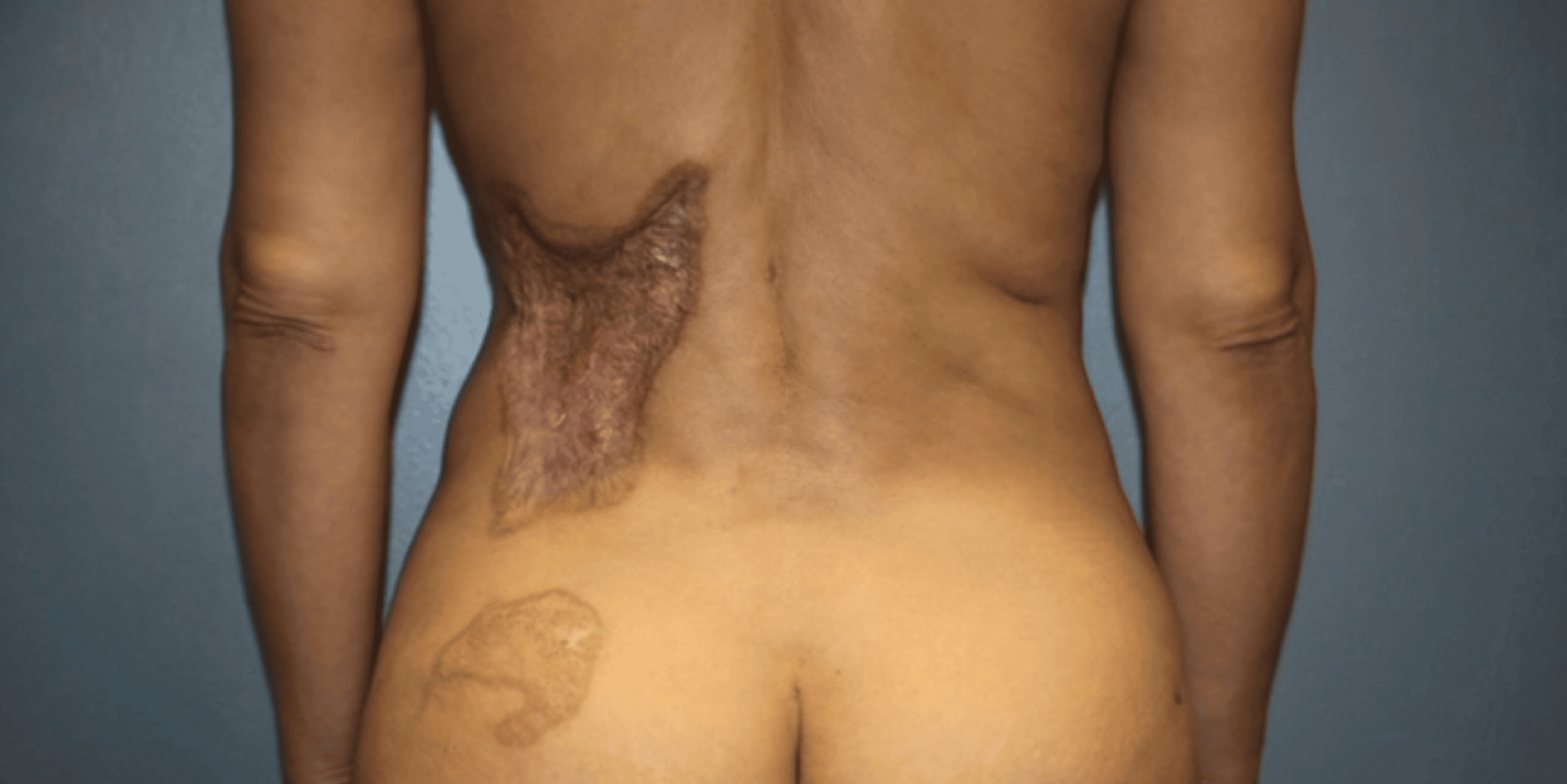
The patient, a 56-year-old female, had a hypertrophic, contracted burn scar measuring 20 x 10 cm in total surface area located on her left flank and posterior trunk following an iatrogenic saline hot pack injury two years prior.

On examination, a scar measuring 20 cm x 10 cm was located over the patient's left flank and posterior trunk (Figure 1). It was hypertrophic in areas, firmly adherent to underlying tissue, and had caused a mild contracture affecting the surrounding skin mobility and resulting in a contour deformity. There was an obvious textural and pigmentary mismatch between the grafted scar area and the adjacent normal skin. Given the size and location of the scar, simple excision and direct closure were not feasible without causing excessive tension. We discussed reconstructive options with the patient, including serial excisions versus the use of tissue expanders. We recommended tissue expansion to recruit adequate nearby skin for a more anatomical reconstruction, avoiding the need for additional grafting. The patient was counseled on the staged nature of treatment, the time commitment, and potential complications such as infection or expander exposure. The patient elected to proceed with our proposed staged approach using tissue expanders, followed by secondary scar revision techniques (laser and needling) for optimal results.

The management proceeded in several stages over the course of approximately two years.

Stage 1: tissue expander placement (May 2022)

Reconstruction began with silicone tissue expander placement during the initial surgery. Under general anesthesia, a horizontal incision was made over the burn scar and deepened down to the level of the muscular fascia. A pocket was created in the left central back region and irrigated with antibiotic solution, saline, and Betadine. Based on the size, shape, and location of the pocket, a curved, kidney-shaped 750-1200 mL tissue expander with an integrated port was chosen and placed and secured to the thoracolumbar fascia. Once this expander was in place, a second pocket was created in the left lower flank using the same incision site to allow for open-end placement of a curved 150-350 mL tissue expander with a remote port. This port was placed superficially in front of the rib cage for suitable outpatient access. After irrigation of both pockets, meticulous hemostasis was ensured. Subsequently, a complex surgical site closure was performed with 2-0 polydioxanone (PDS) sutures anchored to the fascial layer, allowing for separation of the two tissue expanders. There were no intraoperative complications.

Stage 2: expansion period (June-July 2022)

Expansion of the implants was initiated three weeks after placement and continued on a bi-weekly schedule. Saline was incrementally injected into each expander through the fill ports during office visits. Over approximately eight weeks, the upper expander was gradually inflated to about 700 mL and the lower expander to about 200 mL, achieving sufficient tissue stretch. The expansion was guided by tissue tolerance. Each session was stopped if the patient experienced excessive tightness or blanching of the overlying skin. The patient experienced mild discomfort during expansions but no significant pain or skin compromise. By the end of this phase, the surrounding skin had expanded appreciably, providing a redundancy of healthy tissue over and around the scar.

Prior to the patient's scar revision surgery, she had one non-ablative laser therapy session in August 2022 using Nd:YAG 1064 nm and standard settings for conservative scar rejuvenation: 4J, 0.3 ms, 8 mm, 8 Hz (excel V, Cutera, Brisbane, CA, USA).

Stage 3: tissue expander removal, scar excision, and closure with expanded skin (October 2022)

Five months after tissue expander placement, the patient returned to the operating room for tissue expander removal, scar excision, and definitive reconstruction. The expanded skin flaps were elevated, and the entire burn scar, including the previous skin graft and underlying scar tissue, was excised. Given the large expanse of tissue generated by the two expanders, we were able to mobilize the adjacent skin flaps medially to primarily cover the defect. The two expanded flaps were advanced and brought together under minimal tension, resulting in a T-shaped closure in the back. All tissue expanders and ports were removed during this procedure. Two Jackson-Pratt drains were placed. The incisions were closed in layers using deep absorbable sutures and a running nylon skin suture. Pathology of the excised scar confirmed fibrous scar tissue with no malignancy. There were no intraoperative complications, and the patient recovered uneventfully. Both drains were removed after a week.

Stage 4: adjunctive scar therapies (November 2022-April 2024)

Following her second surgery, the patient underwent multiple laser therapy and microneedling sessions as well as one office-based scar revision. Specifically, three 1064 nm Nd:YAG sessions using standard settings were performed at seven, ten, and thirteen weeks postop (November 2022 - January 2023). This was followed by three microneedling sessions (0.5-1.0 mm) over eight weeks (May-June 2023). Persistent hypertrophy measuring 6x3 cm at the superior margin of the surgical site was excised under local anesthesia in the office, and a complex layered closure was done without complications (August 2023). Subsequently, the patient received three more laser therapy sessions in August 2023, November 2023, and April 2024. The patient also had microneedling done during her November 2023 office visit.

Outcome

**Figure 2:**
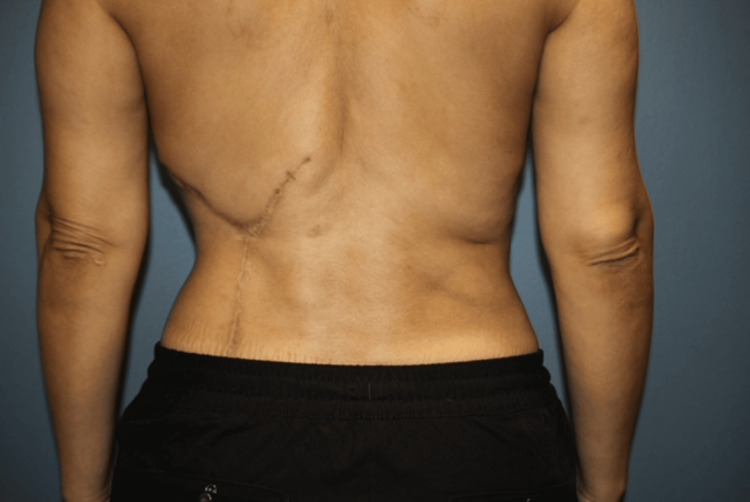
Final outcome after 23 months of staged scar revision showing improved contour, elimination of the previous patch-like hypertrophic graft scar, and a well-healed revision scar.

After staged treatment over the course of 23 months, the patient had significant contour restoration and elimination of patch-like hypertrophic scar tissue. Successive laser/microneedling sessions made her revision scar flatter and softer with improved pigment blend. The patient reported substantially improved body image and was very satisfied with the results (Figure 2). Objective scar assessment was performed with the Modified Vancouver Scar Scale (MVSS) [13] at baseline and late follow-up at 23 months using standardized photographs and clinical examination: the total score decreased from ~10 to ~3, driven by improvements in pigment, vascularity, pliability, and height.

## Discussion

Tissue expansion for large grafted trunk scars

When color/texture mismatch and contour deformity are the chief complaints, tissue expansion provides adjacent, like-matched skin with intact vascular and neural supply. Regrafting or distant flaps often cannot replicate these advantages on broad trunk surfaces. Post-burn series report high rates of successful reconstruction with acceptable complication profiles when technique and follow-up are optimized, including low rates of infection or device exposure in contemporary cohorts [5-7]. Our patient’s course (two expanders, ~8 weeks of inflation, then complete scar excision and tension-free primary closure without expander-related complications) is consistent with these outcomes and demonstrates how expansion can replace a conspicuous grafted patch with native skin and restore flank/back contour [5-7].

Long-pulsed 1064-nm Nd:YAG laser (vascular-targeting)

Hypertrophic scars are hypervascular and pro-inflammatory. Non-ablative long-pulsed Nd:YAG has documented benefit for hypertrophic/keloid scars, improving thickness and erythema with favorable tolerability [9]. The 1064-nm wavelength penetrates more deeply than the pulsed-dye laser, enabling photocoagulation of reticular dermal vessels and down-regulation of angiogenic signaling; clinical series and prospective cohorts show improvements in thickness, erythema/vascularity, pliability, and symptoms with favorable tolerability [8-10]. Recent series also support Nd:YAG reducing early scar redness and optimizing remodeling when used in staged protocols [10]. 1064-nm Nd:YAG was chosen over 532-nm Nd:YAG due to its greater effect on deeper blood vessels and darker lesions [14]. Nd:YAG has also been seen to have a lower risk for atrophic scars and hypopigmentation compared to alternate lasers (krypton, copper bromide, and argon) [15]. In our pathway, interval and post-closure Nd:YAG sessions targeted vascular components and texture with minimal downtime, complementing expansion rather than replacing it.

Microneedling (percutaneous collagen induction)

Increasing evidence supports microneedling for hypertrophic scars, with controlled micro-injury stimulating remodeling of disorganized collagen and elastin as well as melanocyte redistribution while preserving epidermal integrity and improving pliability and surface texture. Prospective and randomized data in post-burn hypertrophic scars demonstrate significant reductions in validated scar scores (e.g., VSS/MVSS [13]) over sequential sessions and across skin types [11,12]. In this case, three microneedling sessions (0.5-1.0 mm) plus one combined laser-microneedling visit produced additional flattening/softening and pigment blending after the contour was normalized surgically. Our patient’s subjective and clinical improvements matched with expected effects, and the modality was well tolerated.

MVSS

MVSS remains among the most widely reported burn-scar scales in clinical practice. It is pragmatic at the point of care and responsive in the domains most affected by our interventions (vascularity, height, pliability, and pigment) [13]. Objective scoring aligns with better patient-reported outcomes as scar quality improves [16], supporting both reproducibility and counseling. We applied MVSS at baseline and late follow-up using standardized photographs and clinical examination. The magnitude of improvement we observed with MVSS (decreasing from ~10 at baseline to ~3 at 23 months) is in line with reported reductions after vascular lasers and microneedling, particularly when these modalities are deployed sequentially following tension-free reconstruction [8-12]. The domain-level gains (vascularity/height/pliability/pigment) map directly to the mechanisms of Nd:YAG (vascular) and microneedling (dermal remodeling), reinforcing the internal consistency of the treatment pathway. 

Clinical takeaway

For extensive grafted trunk scars, a stepwise pathway is reproducible and low-morbidity: (1) replace the graft with like-matched, sensate skin via expansion to correct contour; (2) modulate scar biology with long-pulsed 1064-nm Nd:YAG; and (3) remodel collagen with microneedling. Judicious office scar excision can address focal residual hypertrophy. Outcome tracking with MVSS documents change and supports comparability to published results, and strengthens counseling and quality assurance.

## Conclusions

Iatrogenic hot-pack burns can cascade into infection, grafting, and disfiguring scars. In extensive grafted trunk scars, tissue expansion enables complete scar replacement with adjacent, like-matched skin and reliable contour restoration. Adjunct 1064-nm Nd:YAG laser (vascular targeting) and microneedling (collagen remodeling) further improve MVSS domains (pigment, vascularity, pliability, height) with low downtime. In this case, MVSS improved from ~10 to ~3 at 23 months, matching the patient’s functional and psychosocial gains. A structured, outcomes-tracked pathway like this is supported by multi-study experience and is feasible to reproduce in similar presentations.
